# Editorial: Evolution, biochemistry and function of the dystroglycan-axis: from human diseases to model organisms

**DOI:** 10.3389/fmolb.2025.1712184

**Published:** 2025-10-13

**Authors:** Aaron M. Beedle, Mohammed Akaaboune, Bulmaro Cisneros, Andrea Brancaccio

**Affiliations:** ^1^ Faculty of Pharmaceutical Sciences, University of British Columbia, Vancouver, Canada; ^2^ Department of Molecular, Cellular, and Developmental Biology, Ann Arbor, MI, United States; ^3^ Departamento de Genética y Biología Molecular, CINVESTAV Zacatenco IPN, Ciudad de México, Mexico; ^4^ School of Biochemistry, University of Bristol, Bristol, United Kingdom; ^5^ Institute of Chemical Sciences and Technologies “Giulio Natta” (SCITEC) CNR, Rome, Italy

**Keywords:** dystroglycan, extracellular matrix, basement membranes, protein domains and motifs, mutants, dystroglycanopathies, protein trafficking, glycosylation

Over the last 30 years, the field of dystroglycan (DG) research has garnered significant attention due to its critical role as a non-integrin adhesion complex that bridges the extracellular matrix to the cytoskeleton. The DG adhesion complex is fundamental both during development and in adulthood. It is composed of two noncovalently interacting subunits arising from the cleavage of a single product: the extracellular and heavily glycosylated α-DG, which binds with high affinity to extracellular matrix partners, and the transmembrane β-DG, whose cytodomain includes a dystrophin binding site. DG, the core of the larger dystrophin-glycoprotein complex, provides a fundamental link between the extracellular matrix and the intracellular cytoskeleton.

The involvement of α-DG in severe neuromuscular diseases, such as Walker-Warburg Syndrome (WWS), Muscle-Eye-Brain (MEB) disease, and autosomal recessive Limb-Girdle muscular dystrophy type 2P, underscores its importance in biomedicine. As outlined by the studies of Esapa et al. and Sciandra et al., several recent works have expanded our understanding of DG biochemistry, structure, and function, revealing its potential as a target for personalized therapeutic approaches.

Based on these premises, substantive work is necessary to further unravel the molecular aspects of the DG complex that remain elusive, hindering our full comprehension of its pathophysiological and biochemical role in cells and tissues.

Sugar moieties forming the unusual α-DG glycan structure play a crucial role for its function by working as a “scaffold” (also known as *matriglycan*) that is recognized by laminins and other extracellular binding partners. A series of enzymes, mostly glycosyltransferases located in the endoplasmic reticulum and Golgi, are responsible for the correct post-translational modification of α-DG. Genetic alterations of the corresponding genes cause the “secondary dystroglycanopathies”, due to the loss of α-DG functional glycans, highlighting the importance of a full molecular characterization of these enzymes to elucidate their mechanism of action.

The first study in this Research Topic, an original research work by Esapa et al. is focused on one of these important enzymes, FKRP (Fukutin-related protein), which is a ribitol phosphotransferase. Intracellular dynamics and structural consequences of a panel of missense mutations affecting the FKRP protein have been analysed using a range of different techniques, showing that mutations significantly affect the overall aggregation state of the protein altering disulfide bridges and folding. Using fluorescence recovery after photobleaching, it was shown that the intracellular mobility of most FKRP mutants is significantly impaired but can be rescued by reducing agents. Mass spectrometry was used to show that wild-type and mutant FKRP differentially associate with several endoplasmic reticulum (ER)-resident chaperones, whereas structural modelling was employed to show that disease-associated FKRP missense mutations affected the local environment of the protein. Overall, this study strongly reinforces the notion that protein misfolding contributes to the molecular pathophysiology of FKRP-deficient muscular dystrophies, opening possible therapeutic avenues based on molecules that rescue this folding defect.

In the second contribution, a mini-review by Sciandra et al. investigates how the DG complex is involved in both adhesion and signalling. The timely review explores the capability of DG to form or belong to, different sub-complexes in different tissues such as skeletal muscle, the nervous system, the retina and others. It then focuses on a limited amount of works suggesting that DG can behave as a mechano-transducing receptor, with particular emphasis on innovative Research Topic such as the possible role played by the agrin–DG–YAP interaction in cardiac muscle regeneration, or the intriguing role proposed for β-DG in the nucleus.

An intriguing mini-review by Katz and Diskin addresses the importance of α-DG glycosylation and its role in infectious diseases such as viral infections caused by arenaviruses. In fact, it is well-known that Lassa virus (LASV) targets the long linear polysaccharide *matriglycan* protruding from the α-DG *core protein* to enter cells. A particular focus is dedicated to recent structural data on LARGE1 and on the LASV spike complex collected in the two authors’ laboratories, offering novel structural insights into LARGE1 synthesis of *matriglycan*.

We believe that both mini-reviews are of strong appeal to the wider DG-oriented scientific community, as they deal with numerous subjects related to the role of the DG-axis that are still relatively unexplored, despite having an impact in several branches of biomedicine.

The final contribution in this Research Topic, an original research paper by Sciandra et al. reported on how a panel of DG missense mutants (analyzed *versus* the wild-type protein) can affect the post-translational trafficking and localization of the mature complex. Collecting a series of microscopic images at super-resolution in live transfected cells, enabled the authors to show the presence of some significant differences in the subcellular pattern of localization in different cell lines, that could be used to predict functional consequences of a mutant protein. The crucial take-home message in this case being that, for an accurate analysis of primary dystroglycanopathy-causing mutations to have potential diagnostic/prognostic value, a panel of different cells should be employed, rather than a single cell line.

While the DG-field has profited from recent novel structural data collected through 3D cryo-electron microscopy (cryo-EM) analysis. Our overall impression is that there is still a significant lack of knowledge on many evolutionary, biochemical and functional details concerning the DG complex. In this respect, our Research Topic could be regarded as an early milestone in the path to establishing additional new investigative approaches and models, particularly from laboratories so far working “only at the periphery” of the DG complex. Once again, we wish to stress the relevance that establishing multidisciplinary research approaches could have for solving a series of questions still open over the biological role of the DG-axis.

